# Optical genome mapping detects cryptic high‐risk and targetable abnormalities in adult AML

**DOI:** 10.1111/bjh.70349

**Published:** 2026-02-01

**Authors:** Audrey Bidet, Elodie Laharanne, Manon Dos Santos, Anne‐Charlotte De Grande, Thibaut Leguay, Arnaud Pigneux, Eric Frison, Sandrine Achard, Stéphanie Dupont‐Moufakkir, Emilie Klein, Pierre‐Yves Dumas

**Affiliations:** ^1^ CHU Bordeaux, Service d'Hématologie Biologique Bordeaux France; ^2^ CHU Bordeaux, Service d'Oncologie et d'Hématologie Pédiatrique Bordeaux France; ^3^ CHU Bordeaux, Service d'Hématologie Clinique et de Thérapie Cellulaire Bordeaux France; ^4^ Université de Bordeaux, Inserm, UMR1312, BRIC, BoRdeaux Institute of onCology Bordeaux France; ^5^ CHU Bordeaux, Service d'Information Médicale Bordeaux France

**Keywords:** acute myeloid leukaemia, optical genome mapping, prognosis, risk stratification

## Abstract

Acute myeloid leukaemia (AML) risk stratification relies on cytogenetic and molecular abnormalities defined by European LeukemiaNet (ELN) 2022. Conventional cytogenetic techniques, including chromosomal banding analysis (CBA) and fluorescence in situ hybridization, have limited resolution and may miss cryptic events. Optical genome mapping (OGM) is a genome‐wide approach capable of detecting balanced and unbalanced structural variants with high resolution, potentially revealing cryptic abnormalities of diagnostic and prognostic relevance. We retrospectively studied 100 adults with newly diagnosed AML, each showing one to two cytogenetic abnormalities and lacking the World Health Organization 2022–defining rearrangements or baseline ELN adverse karyotypes. OGM was performed to evaluate additional cytogenetic abnormalities and impact on ELN 2022 risk classification. Clinical outcomes were explored descriptively. OGM detected 91.4% of abnormalities identified by CBA and provided additional information in 37% (95% confidence interval: 28%–47%) of patients. Fourteen per cent was reclassified to an unfavourable cytogenetic group, and 7.7% was reclassified to ELN 2022 adverse risk. Cryptic *KMT2A* and *NUP98* lesions were found in 10% of cases, highlighting potential therapeutic targets. Survival analyses suggested a trend towards poorer outcomes in patients reclassified as adverse, though the small sample limits definitive conclusions. In low‐complexity AML, OGM provides substantial incremental diagnostic value, detecting cryptic high‐risk and targetable abnormalities, supporting its use as a complementary tool.

## INTRODUCTION

Despite major advances in genomic profiling and therapeutics, acute myeloid leukaemia (AML) remains associated with poor outcomes in patients. Prognostic classifications such as European LeukemiaNet (ELN) 2022,^1^ used to guide therapeutic choices, are largely based on chromosomal abnormalities (CAs).^2^ CAs are currently evaluated mostly using conventional cytogenetic techniques, including chromosomal banding analysis (CBA) and fluorescence in situ hybridization (FISH), with more limited use of array‐based or sequencing approaches. Optical genome mapping (OGM) has emerged as a promising genome‐wide, amplification‐free technology for the detection of structural variants (SVs) in AML. A pivotal study by Gerding et al.^3^ demonstrated a high concordance with conventional cytogenetics, while revealing additional cryptic structural abnormalities not detected by standard approaches, thereby establishing the diagnostic relevance of OGM in AML.

A recent review by Nilius‐Eliliwi et al.^4^ highlighted unresolved issues regarding cytogenetic complexity and its interpretation within the ELN 2022 framework. While the adverse prognostic significance of complex karyotypes (CKs) and monosomal karyotypes (MKs) is well established, translating OGM‐detected complexity into ELN 2022 categories presents new challenges. These considerations underscore the need for real‐world clinical studies evaluating the added value and limitations of OGM in selected AML populations. To address this, we focused on patients with one to two cytogenetic abnormalities, deliberately excluding the World Health Organization (WHO) 2022–defining rearrangements and baseline ELN adverse karyotypes.

In this context, we aimed to evaluate the proportion of AML cases with additional CAs detected by OGM compared with standard cytogenetics, focusing on low complexity karyotypes representing a diagnostic grey zone, and to explore the impact of OGM on ELN 2022–based risk reclassification and to descriptively assess associated clinical outcomes.

## MATERIALS AND METHODS

### Patients

This single‐centre retrospective study involved patients with newly diagnosed AML according to the WHO 2022 criteria^5^ between 1 January 2010 and 31 December 2022 included in the DATAML Bordeaux Registry. Eligible patients were aged over 18, had a bone marrow sample available and a karyotype showing one or two cytogenetic abnormalities, excluding recurrent WHO 2022 abnormalities^5^ such as t(15;17)(q24;q21.2)/*PML::RARA* or other RARA rearrangements, t(8;21)(q22;q22.1)/*RUNX1::RUNX1T1*, inv.(16)(p13.1q22) or t(16;16)(p13.,1;q22)/*CBFB::MYH11*, t(9;11)(p21.3;q23.3)/*KMT2A::MLLT3* or other KMT2A rearrangements, t(6;9)(p22.3;q34.1)/*DEK::NUP214*, inv.(3)(q21.3q26.2) or t(3;3)(q21.3;q26.2)/*GATA2, MECOM* (*EVI1*) or other MECOM rearrangements and t(9;22)(q34.1;q11)/*BCR::ABL1*. Patients who initially presented with an unfavourable karyotype according to ELN 2022 were excluded (monosomy 5 or del(5q), monosomy 7, monosomy 17 or abnormal 17p, CK and MK).^1^ This selection was designed to assess the incremental diagnostic value of OGM specifically in cases where conventional cytogenetics is non‐discriminatory, thereby avoiding the reiteration of established prognostic categories and focusing on situations in which OGM could meaningfully influence diagnostic interpretation. Among the 105 patients initially selected, five were excluded because DNA extracted from cell pellets did not meet the quality thresholds required for OGM analysis. Among them, 91 received intensive chemotherapy (ICT), mainly with daunorubicin (60–90 mg/m^2^, 3 days) and cytarabine (100–200 mg/m^2^, 7 days). Next‐generation sequencing (NGS) mutation analysis was performed for ELN 2022–based risk stratification.^1^ The study complied with the Declaration of Helsinki was approved by national regulatory authorities (ClinicalTrials.gov Identifier: NCT05499611). DATAML is approved by French authorities [CNIL (N°915 285), CCTIRS (N°15.319)].

### Cytogenetics

CBA with R‐banding was performed on diagnostic bone marrow aspirates after 24 h of short‐term culture. At least 20 metaphases were analysed, and karyotypes were described using the International System for Human Cytogenetic Nomenclature.^6^ The definitions of CK and MK followed the ELN 2022 criteria^1^: CK is defined as ‘three or more unrelated CAs’ in the absence of recurrent translocations or inversions (t(8;21), inv.(16) or t(16;16), t(9;11), t(v;11)(v;q23.3), t(6;9), inv.(3) or t(3;3), t(9;22)). Furthermore, CK does not include cases with hyperdiploid karyotypes without structural CAs; MK corresponds to two or more autosomal monosomies or a single autosomal monosomy combined with at least one structural CA (excluding markers, rings and core binding factor abnormalities).

### OGM

Ultra‐high molecular weight DNA was extracted using the ‘Blood and Cell Culture DNA Isolation Kit’ as per the manufacturer's protocol, with modifications for resuspending cells from 62 dimethylsulphoxid and 43 cell pellets, as recommended by Bionano Genomics. After quantification with a Qubit fluorometer (Invitrogen, France), 750 ng of DNA per sample was labelled using the Bionano Prep DLS labelling kit. Samples were processed on Bionano Saphyr chips for linearization and imaging, and analysed with Access 1.7.2 and the rare variant analysis pipeline, aligned to the hg38 reference. Most samples achieved a coverage of at least 300×, and only those meeting quality criteria (N50) were considered. Five cell pellet samples were excluded at this stage. The analysis first focused on ‘macroscopic’ abnormalities—those detectable by karyotyping and used in current prognostic classifications: that is, balanced abnormalities, unbalanced abnormalities >5 Mb, submicroscopic structural abnormalities between 500 kb and 5 Mb relevant to AML, copy number variations (CNVs) over 500 kb and rearrangements resembling chromoanagenesis. Only these CAs were considered when defining CKs or MKs, as was also the case in the cost–benefit analysis.

In a second step, all abnormalities involving a predefined set of 109 genes and 8 AML‐associated regions (‘AML bed file’), derived from Levy et al.^7^ and enriched with additional genes relevant in AML, were considered irrespective of size, provided they met confidence thresholds recommended by the manufacturer (Table S1).

SVs were identified with a precision of 25–50 kb and CNVs of at least 10 kb, as per Levy et al.^7^ This method does not detect point mutations or minor changes (<15%). Centromeric repeats, satellite sequences and unlabelled regions were excluded from the study. Only variants absent from the Bionano Solve 3.6 database of human controls were retained. Discordant cases or cryptic CAs were validated by FISH using appropriate probes when available or polymerase chain reaction (PCR) for *DEK::NUP214* transcript or *KMT2A‐* partial tandem duplication (*KMT2A*‐PTD).

Although the study was retrospective and spanned several years, heterogeneity was minimized through a uniform re‐evaluation of conventional cytogenetic results, centralized OGM analysis in a single laboratory and the application of consistent quality and interpretation criteria across all samples.

### FISH

Cytogenetic preparations were placed on superfrost slides, hybridized following the manufacturer's protocol (Calibre Scientific‐Amplitech, France) and analysed with a Zeiss fluorescence microscope, using Metasystems software.

### PCR

*DEK::NUP214* and *KMT2A*‐PTD transcripts were assessed by qualitative reverse transcription polymerase chain reaction. Total RNA from diagnostic bone marrow was reverse‐transcribed. For *DEK::NUP214*, PCR was performed with primers in *DEK* exon 2 and *NUP214* exon 9 under standard cycling conditions.^8^ Products were visualized by agarose gel electrophoresis, with positive/negative controls in each run. *KMT2A*‐PTD was screened according to Schnittger et al.^9^ using primers in *KMT2A* exons 8 and 3, amplifying the duplication‐specific fragment. Amplicons were analysed by agarose gel and confirmed by repeat PCR and Sanger sequencing.

### NGS

Targeted NGS of 58 genes recurrently mutated in myeloid neoplasms was performed at diagnosis to support ELN 2022 molecular risk stratification.^1^ The panel covered ELN‐relevant genes, including myelodysplastic syndrome (MDS)‐related genes (*ASXL1, BCOR, EZH2, RUNX1, SF3B1, SRSF2, STAG2, U2AF1, ZRSR2*) and *NPM1, FLT3, CEBPA* and *TP53*, as well as additional genes of diagnostic or therapeutic relevance in AML (e.g. *IDH1/2* and *RAS* pathway genes). Detailed methods are provided in Table S2.

### Statistical analysis

Based on the first largest study conducted solely on AML,^7^ we assumed that 15%–20% of participants would have at least one additional CA, regardless of their prognostic impact, detected by OGM. We initially planned to include 120 participants, based on feasibility and sufficient precision of the estimate for the primary end‐point (width of the 95% confidence interval [95% CI] of 13%–15%). We were finally able to identify 105 eligible cases for the study.

Continuous variables are presented as median (range) and categorical variables as counts (percentages). Overall survival (OS) and event‐free survival were estimated by the Kaplan–Meier method and compared using the log‐rank test, according to the ELN 2022 criteria and to the number of abnormalities present in the predefined set of 109 genes and eight regions of interest in AMLs. Cox proportional hazards models were used to estimate hazard ratios (HRs) and 95% CIs. Multivariable analysis included clinically relevant covariates selected a priori (age as a continuous variable, and key molecular markers *NPM1* and *FLT3‐*ITD) together with the binary variable ‘OGM reclassified as ELN adverse risk’. Given the limited number of events and small subgroup sizes, model parsimony was enforced (maximum 2–4 covariates). When standard Cox models were at risk of small‐sample bias, bootstrap resampling (500 iterations) was used as a sensitivity analysis to derive empirical CIs. Two‐sided *p*‐values <0.05 were considered statistically significant. Analyses were performed using R version 4.2.3 and Python (statsmodels).

## RESULTS

### Study population, karyotype and NGS

This study included 100 patients, with a median age of 63 years (range: 19–80 years, inter‐quartile range [IQR]: 55–63) and a male‐to‐female ratio of 1.77. Among the 91 patients treated by ICT, the median age was 61 years (range 19–78 years, IQR: 52–68) and the male‐to‐female ratio was 1.93. Table 1 provides the demographic and biological characteristics of both groups. According to the ELN 2022 classification, all patients had karyotypic abnormalities that prevented them from being immediately classified as having a favourable or adverse risk profile. In ICT patients, the most frequent mutations were *NPM1* (*n* = 26), *FLT3*‐ITD (*n* = 22), *CEBPA* basic leucine zipper domain (bZIP) in frame (*n* = 6), *TP53* (*n* = 2) and MDS‐related gene alterations (*n* = 37). Based on ELN 2022 cytogenetic and molecular criteria, 22 patients (24%) were classified as favourable, 33 (36%) as intermediate and 36 (40%) as adverse risk.

**TABLE 1:** Patients characteristics.

	All (*n* = 100)	ICT (*n* = 91)
Demographics and clinical variables		
Age (years), median (min–max)	63 (19–80)	61 (19–78)
Female *n* (%)	36 (36)	31 (34)
WBC (10^9^/L), median (IQR)	54 (1.3–359)	31 (1.3–359)
Platelets (10^9^/L), median (IQR)	57 (10–451)	57 (10–451)
De novo AML *n* (%)	76 (74.5)	73 (80.2)
Gene mutation		
*NPM1, n* (%)	26 (26)	26 (28.6)
*CEBPA* (*in frame Bzip*) *n* (%)	7(7)	6 (6.6)
*FLT3‐*ITD *n* (%)	23 (23)	22 (24.2)
*FLT3‐*TKD *n* (%)	8 (8)	8 (8.8)
*TP53 n* (%)	2 (2)	2 (2.2)
MDS‐related gene mutation *n* (%)	43 (43)	37 (40.7)
ELN 2022 risk category (karyotype + mutations)		
Favourable *n* (%)	26 (26)	22 (24)
Intermediate *n* (%)	33 (33)	33 (36)
Unfavourable *n* (%)	41 (43)	36 (40)
OGM abnormalities		
CK *n* (%)	11 (11)	10 (11)
MK *n* (%)	2 (2)	2 (2.2)
*DEK::NUP214 n* (%)	1 (1)	1 (1.1)
3q abnormality *n* (%)	1 (1)	0 (0)
Total patients upgraded	15	13
*NUP98 n* (%)	3 (3)	2 (2.2)
*KMT2A‐PTD n* (%)	7 (7)	7 (7.7)

Abbreviations: CK, complex karyotype; ELN, European LeukemiaNet; ICT, intensive chemotherapy; IQR, inter‐quartile range; Max, maximum; Min, minimum; MK, monosomal karyotype; OGM, optical genome mapping; WBC, white blood cell count.

### Concordance of CBA and OGM

The total number of CAs identified by karyotype was 128. OGM identified 91.4% of the CA detected by CBA and provided additional information in 37 patients (37%, 95% CI: 28–47), either by revealing additional CA or by refining those previously identified on the karyotype (Table S3—UPN1–UPN37).

OGM missed cytogenetic abnormalities detected by CBA in eight patients (8%) (Table S3: UPN38–UPN45). The undetected abnormalities correspond to the known limitations of the OGM, namely subclones in six of eight patients, mostly CNVs (UPN38, 39, 40, 42, 43, 44) with a clone size verified by interphase FISH averaging 9% (7%–12%), tetraploidy in one patient (UPN45) and SVs with breakpoints in centromeric or telomeric regions, which are poorly covered by OGM, in another one (UPN41).

**FIGURE 1:**
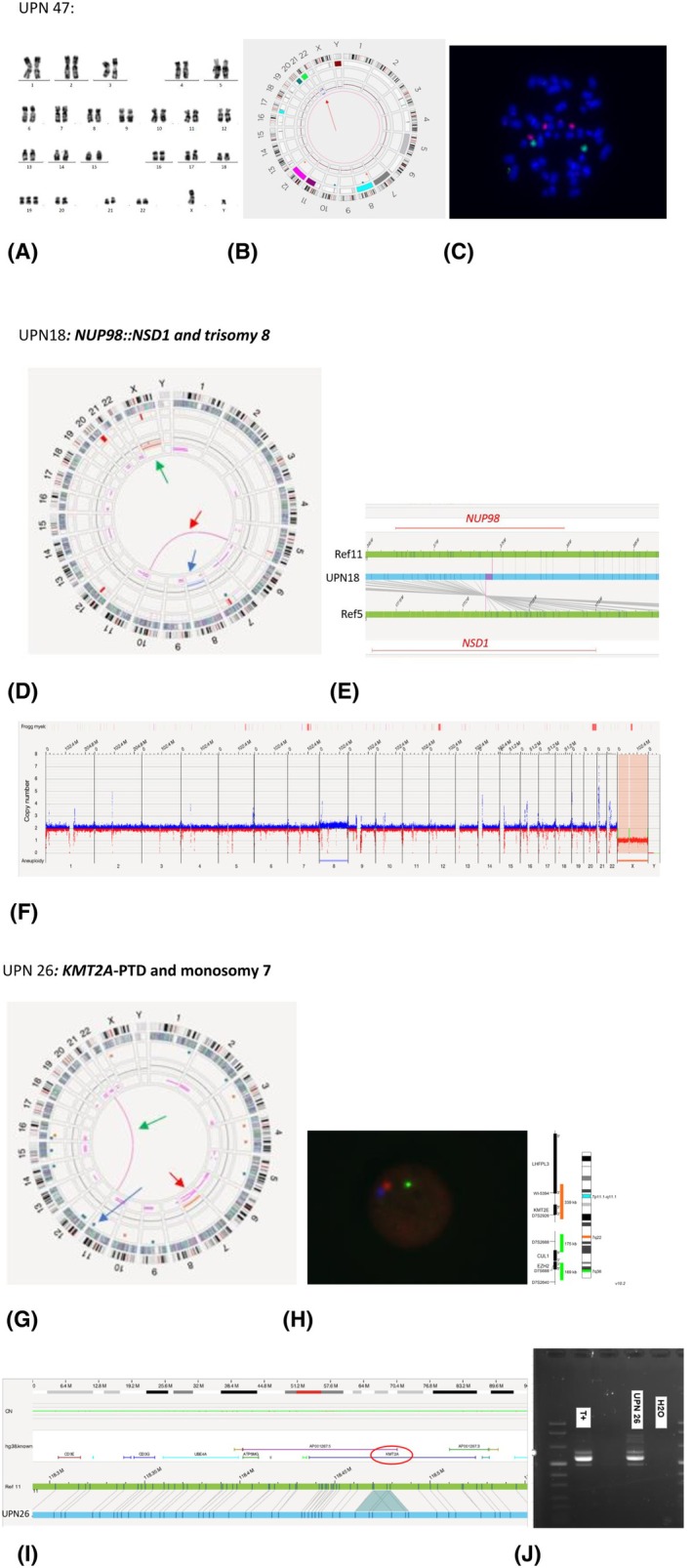
Examples of cytogenetic abnormalities only detected by optical genome mapping (OGM). (A–C) OGM and fluorescence in situ hybridization (FISH) results of UPN47: (A) karyotype suggesting trisomy 19, (B) Circos plot revealing a trisomy 22 (red arrow), (C) FISH analysis using chromosome paint probes (metaSystems) showing three copies of chromosome 22 (red) and two copies of chromosome 19 (green). (D–F) OGM results of UPN18. (D) Circos plot revealing a translocation t(5;11)(q35.3;p15.4) (red arrow) and a trisomy 8 (blue arrow). Monosomy X (green arrow) was seen on the karyotype. (E) Focus on the translocation t(5;11)(q35.3;p15.4) with a breakpoint located in *NSD1* on 5q35.3 and *NUP98* on 11p15.4. (F) Copy number variation (CNV) profile showing a trisomy 8 (blue) and monosomy X (orange). (G–J) OGM, FISH and PCR results of UPN26. (G) Circos plot revealing a monosomy 7 (red arrow) and an insertion at 11q23.3 (blue arrow); translocation t(11;21) (green arrow) was seen on karyotype. (H) Example of a nucleus after hybridization with a triple‐colour 7q. (I) Focus on the insertion at 11q23.3 in *KMT2A* interpreted as *KMT2A* partial tandem duplication. (J) Agarose gel showing a *KMT2A*‐PTD transcript (exons 2–8) comparable to the positive control (T+). [Colour figure can be viewed at wileyonlinelibrary.com]

Of note, in three patients (3%) (Table S2: UPN46–48), the results between CBA and OGM remain contradictory. Both cases also shared a lack of sensitivity of the OGM to detect subclonal abnormalities that could be selected by cell culture. In the first case, FISH confirmed both OGM and CBA findings, that is, trisomy 13 and interstitial deletion in chr.Y, revealing that trisomy 13 was present in only 6% of nuclei, which likely explains why OGM did not detect it. In the second case, the loss of chromosome Y detected by OGM was confirmed by FISH in 16% of nuclei but OGM failed to identify the marker chromosome present in 3/40 mitoses on the karyotype, thus a subclonal CA. In the third case, FISH validated the OGM result for trisomy 22, where chromosome 22 had a large satellite mimicking chromosome 19 (Figure 1A–C).

A median of 1.3 (IQR: 1–4) additional CA were detected by OGM. Five (5%) patients had additional CA belonging to the group of recurrent AML CA: t(5;11)/*NSD1::NUP98* (*n* = 3), t(6;9)/*DEK::NUP214* (*n* = 1) and 3q26.2 anomaly (*n* = 1). Furthermore, monosomy 7 and monosomy 17 were diagnosed in two cases, both resulting in an MK. Ten patients (10%) showed one or two additional abnormalities that led to a reclassification as CK. After considering the cytogenetic abnormalities revealed by OGM, 14 patients (14%) were reclassified as having a karyotype predicting adverse outcomes, not previously detected by CBA (Figure 1D–F). The 3q26 anomaly and the abnormalities leading to CK or MK were all confirmed by FISH.

Ten cases (10%) had an extra anomaly that was either non‐recurrent (per WHO 2022) or did not place the karyotype in the adverse group according to ELN 2022. Seven patients (7%) were found to have *KMT2A* partial tandem duplication (*KMT2A‐*PTD). Recurrent transcripts and *KMT2A*‐PTD were all confirmed by PCR (Figure 1G–J).

In six patients (6%), OGM identified new gene rearrangements, such as *USP36* on der(17) as a new partner for *KAT6B* on der(10) in UPN30. In UPN34, a more complex rearrangement on chromosome 7q led to a *CDK6::MNX1* transcript. Additionally, in UPN37, the translocation t(1;12) resulted in a putative gene fusion *FAM102B::WBP11*. OGM also clarified the structure of derivative and marker chromosomes. For instance, the add(16)(p1?1) in UPN27 was actually a der(16)t(2;16), the ring chromosome (r) in UPN29 was found to be a r(8) and the add(9)(p24) in UPN35 was actually a der(9) harbouring an amplification of 9p and 9q.

### Impact of ELN 2022 classification

**FIGURE 2:**
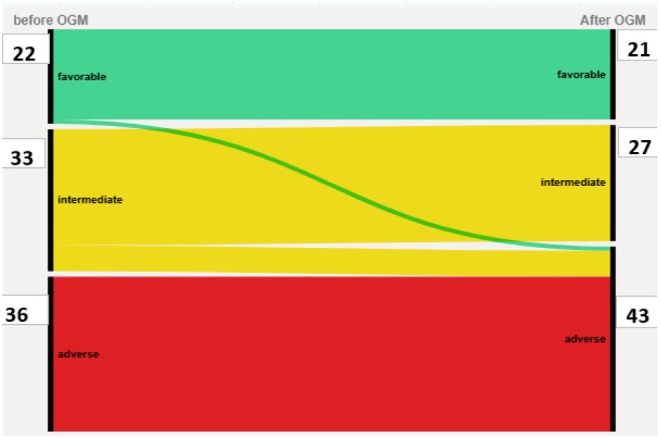
Sankey diagram comparing European LeukemiaNet (ELN 2022) risk before optical genome mapping (OGM) and after OGM. The number of patients in each prognostic group is indicated on the sides of the graph. [Colour figure can be viewed at wileyonlinelibrary.com]

Among the 100 patients, 14 (14%, 95% CI: 8–22) displayed CK, MK, 3q abnormality or *DEK::NUP214*, as refined by OGM, and were thus reclassified as having an unfavourable karyotype according to ELN 2022 (Table 2). We then focused on the ICT subgroup (*n* = 91) according to ELN 2022 recommendations. Within the intensively treated cohort, 33 patients (36%) had at least one additional abnormality detected by OGM, and 12 patients (13.2%, 95% CI: 7–22) were reclassified as having an adverse karyotype. Integrating both cytogenetic and molecular data, seven patients (7.7%, 95% CI: 2–15) were ultimately reclassified into the ELN 2022 adverse prognosis group (Figure 2).

**TABLE 2:** Characteristics of the 14 patients reclassified by the optical genome mapping (OGM) as having an unfavourable karyotype according to European LeukemiaNet (ELN) 2022.

UPN	Karyotype	ELN 2022 before OGM	Additional CA by OGM	Reason for reclassification as unfavourable karyotype	Number of additional ELN abnormalities	ELN 2022 post OGM
2	46,XX,der(7)t(7;13)(q33;q31)[29]/46,XX[1]	Intermediate	ogm[GRCh38] t(6;9)(p22.3;q34.13)(DEK::NUP14)	DEK::NUP214	1	Adverse
4	48,XY,+8,+i(8)(q10)[25]	Favourable	ogm[GRCh38] (12)x3	CK	1	Adverse
7	47,XY,+8[25]	Intermediate	ogm[GRCh38]t(X;Y)(q28;q12), t(5;11)(q35.3;p15.4)(NUP98::NSD1)	NUP98::NSD1 CK	2	Adverse
10	46,XX,add(7)(q22)[12]/46,XX,del(7)(q22q36)[4]/46,XX[4]	Intermediate	ogm[GRCh38] 2p25.3(1615705_3017923)x1,(4)x1,(22)x3; der(7)t(2;7)(p16.2;q22.1)	CK	3	Adverse
13	46,XX,del(7)(q22q36)[20]	Intermediate	ogm[GRCh38] der(7)t(3;7)(q26.2;q21.2)	3 q abnormality	1	Adverse
14	48,XX,+4,+13[8]/46,XX[7]	Adverse	ogm[GRCh38] (19)x1,(20)x1	CK	2	Adverse
15	46,XY,t(6;21)(q21;q22)[12]/45,sl,‐Y[4]/46,XY[4]	Intermediate	ogm[GRCh38] (8)x3, ins(11;?)(q23.3;?)	CK	1	Adverse
18	45,X,‐X[3]/46,XX[17]	Intermediate	ogm[GRCh38] t(5;11)(q35.3;p15.4)(NUP98::NSD1), (8)x3	NUP98::NSD1, CK	2	Adverse
19	46,XY,+1,der(1;7)(q10;p10)[20]	Adverse	ogm[GRCh38] Yq11.23q12(26563807_57212132)x0	CK	1	Adverse
20	46,XX,del(7)(q22q36)[9]/46,sl,del(20)(q12q13)[11]	Adverse	ogm[GRCh38] 5p14.3p13.3(18527423_30713952)x1,13q21.2q21.32(61485268_65630764)x1,18q22.1q22.2(64596530_69799692)x1,(22)x3	CK	4	Adverse
22	47,XY,+8[12]/46,XY[8]	Adverse	ogm[GRCh38] (17)x1	MK	1	Adverse
24	47,XY,+19[10]/48,sl,+13[7]/46,XY[3]	Adverse	ogm[GRCh38] t(4;8)(q34.3;q24.13)	CK	1	Adverse
25	46,XY,+1,der(1;7)(q10;p10)[10]	Adverse	ogm[GRCh38] Yq11.23q12(26551167_57212132)x0,(19)x1	CK	2	Adverse
26	46,XY,t(11;21)(q24;q21)[10]	Intermediate	ogm[GRCh38](7)x1, ins(11;?)(q23.3;?)	MK	1	Adverse

Abbreviations: CA, chromosomal abnormalities; CK, complex karyotype; ELN, European LeukemiaNet; MK, monosomal karyotype; OGM, optical genome mapping.

### Outcomes

We observed a lower median OS (13 months) in this group of patients reclassified by OGM into the ELN 2022 adverse prognosis group, compared to the initial unfavourable prognosis group as per ELN 2022 (19 months, *p* = 0.53). In univariable Cox analysis for OS, OGM reclassification was associated with HR = 1.21 (95% CI: 0.52–2.83). In a multivariable Cox model adjusted for age, *NPM1* and *FLT3‐*ITD, the OS HR for OGM reclassification was 1.71 (95% CI: 0.73–4.03). Bootstrap sensitivity analysis (*n* = 500 resamples) yielded a multivariable HR 95% empirical CI of 0.74–4.94. These estimates indicate a trend towards poorer OS among patients reclassified as adverse by OGM, but CIs are wide due to the small number of reclassified patients.

When we focused on abnormalities relating to the 109 genes and 8 regions associated with AML, the median number of abnormalities (SV and CNV) was 2 (range 0–6) (Table S4). Notably, patients harbouring more than three abnormalities exhibited significantly poorer OS (=405 days) compared with patients with exactly three abnormalities (602 days) or fewer than three abnormalities (872 days; *p* < 0.05) (Figure S1). However, these findings should be interpreted cautiously given the limited sample size and exploratory nature of the analysis.

## DISCUSSION

In this single‐centre cohort of 100 newly diagnosed AML patients, OGM demonstrated high concordance with conventional cytogenetics (91.4%) and provided additional information in over one‐third of cases, leading to adverse cytogenetic group switch in 14% of the whole cohort and to ELN 2022 risk reclassification in 7.7%. These findings support OGM as a complementary tool to current diagnostic approaches, particularly in refining prognostic stratification.

Our results confirm and extend prior evaluations in AML^7^, ^10^, ^11^ by directly linking OGM‐based reclassification to clinical outcomes. Patients reclassified as ELN adverse risk by OGM tended to have lower complete remission (CR)/complete remission with incomplete count recovery (CRi) rates and shorter OS compared with the original adverse risk group. Although multivariable analyses suggested a consistent trend towards poorer outcomes, the limited number of reclassified patients and events limits definitive prognostic conclusions. Accordingly, these analyses should be regarded as exploratory and warrant confirmation in larger, prospective cohorts. Importantly, this study was intentionally enriched for AML cases with low complexity representing a diagnostic grey zone in which conventional cytogenetics may be insufficiently discriminatory. While this design enhances the ability to assess the incremental diagnostic value of OGM, it also limits the generalizability of these findings to patients with clearly favourable or overtly adverse cytogenetic profiles at diagnosis.

Beyond risk reclassification, OGM detected cryptic rearrangements involving *KMT2A* and *NUP98* abnormalities associated with poor prognosis and potential responsiveness to menin inhibitors.^12^ In our series, 10% of patients harboured such lesions, underlining the potential of OGM to facilitate precision medicine approaches. Importantly, OGM integrates in a single assay the detection of balanced and unbalanced rearrangements, large CNVs and submicroscopic events, reducing the need for multiple complementary tests. Both OGM and whole genome sequencing (WGS)^13^ can identify cryptic structural variants that are not detected by conventional cytogenetics. In AML/MDS, WGS has been reported to reveal additional clinically relevant variants in approximately 17–25% of cases, sometimes resulting in changes to ELN risk classification. Nevertheless, each technique has its own limitations. OGM may miss low‐level subclones, centromeric/telomeric abnormalities or tetraploidy and cannot resolve clonal architecture from bulk DNA.^14^ WGS remains difficult to deploy routinely due to its availability, high cost, technical complexity and volume of data generated. It may also miss some complex SVs. These complementary profiles highlight that both technologies could indeed be used to improve AML molecular landscape exploration, although practical considerations and specific detection strengths differ.

Finally, as noted in recent reviews,^4^ OGM higher resolution raises questions regarding the definition and prognostic interpretation of CK and MK. In this study, we considered only abnormalities detectable by conventional karyotyping or recurrent lesions involving ELN 2022–listed genes. The prognostic impact of additional submicroscopic variants remains unknown, and future studies will be needed to refine CK/MK definitions in the OGM era.^6^, ^15^, ^16^

Taken together, our results support the integration of OGM into the initial AML diagnostic workup as a complementary tool to conventional cytogenetics, particularly in cases with intermediate karyotypes. By uncovering cryptic adverse risk abnormalities, OGM may contribute to a more comprehensive cytogenetic assessment and support risk‐adapted decision‐making in selected clinical contexts.

## AUTHOR CONTRIBUTIONS

Conceptualization: A.B. and P.‐Y.D.; software: A.B., E.L. and E.K.; investigation: A.B., M.D.S., A.‐C.D.G., T.L., A.P. and P.‐Y.D.; data curation: A.B., E.L., E.K. and M.D.S.; methodology: A.B., P.‐Y.D. and E.F.; Formal analysis: E.L., M.D.S., S.A. and S.D.‐M.; writing, review and editing: A.B., E.L., M.D.S., A.‐C.D.G., T.L., A.P., E.F., S.A., S.D.‐M., E.K. and P.‐Y.D. All authors have read and agreed to the published version of the manuscript.

## FUNDING INFORMATION

This work was supported by Grants from Bordeaux University Hospital and from region of Nouvelle‐Aquitaine.

## CONFLICT OF INTEREST STATEMENT

The authors have no conflicts of interest to disclose.

## Supporting information


**Figure S1.** Survival according to the number of genomic abnormalities (SV + CNV) detected in the acute myeloid leukaemia (AML) bed file.

**Table S1.** Acute myeloid leukaemia (AML) bed file set of 109 myeloid genes and 8 regions of interest.

**Table S2.** Targeted next‐generation sequencing of 58 genes recurrently mutated in myeloid neoplasms.

**Table S3.** Discrepancies between karyotype and optical genome mapping (OGM) and prognostic impact.

**Table S4.** Number and type of abnormalities, focused on ‘acute myeloid leukaemia (AML) bed file’.

## Data Availability

The data that support the findings of this study are available from the corresponding author upon reasonable request.
