# Supplementary figures and images for: Optical genome mapping detects cryptic high‐risk and targetable abnormalities in adult AML
Source: Br J Haematol. 2026 Feb 1;208(4):1232–9. doi: 10.1111/bjh.70349 (PMC13071487; doi:10.1111/bjh.70349)

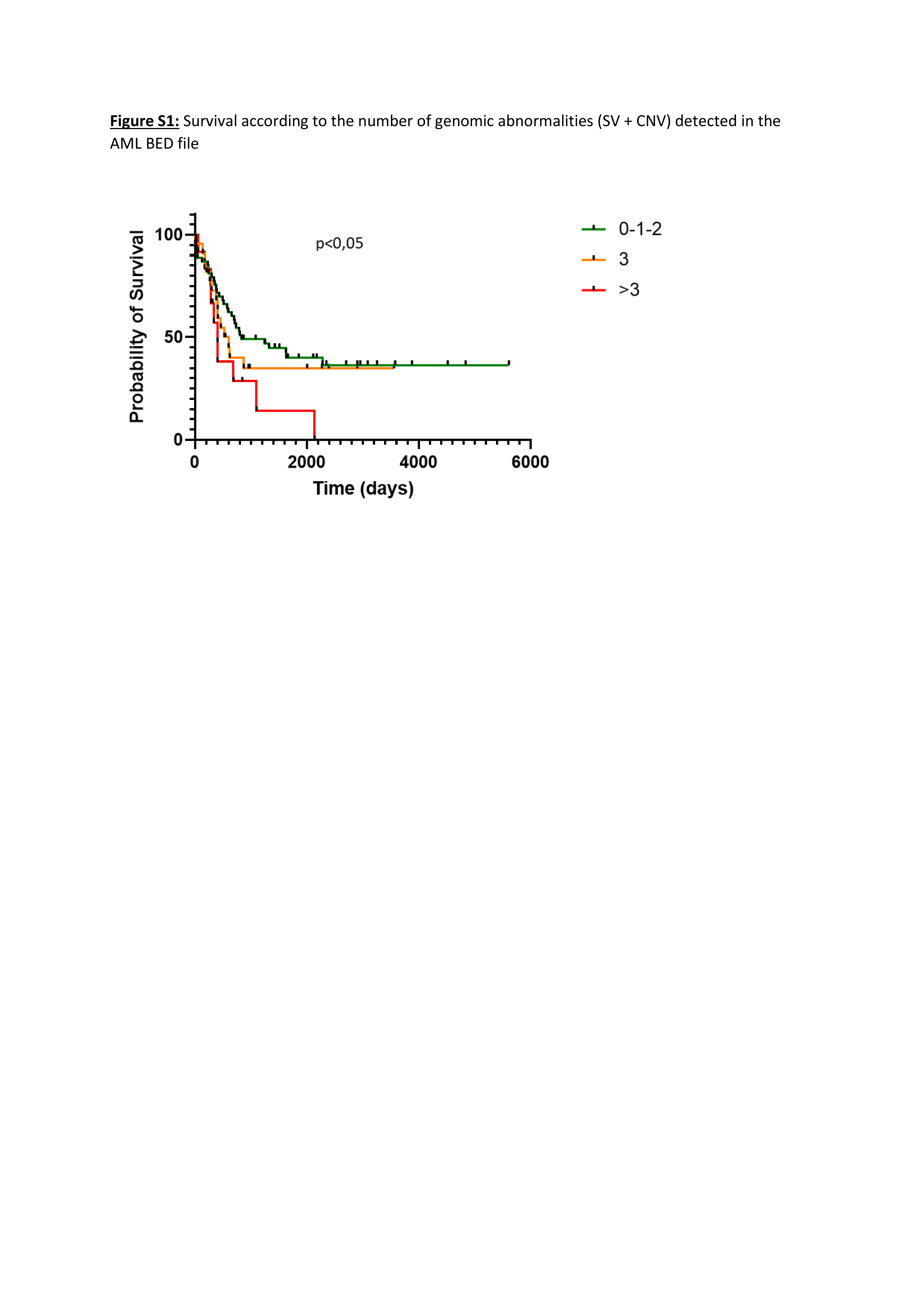

Supplement: Supplementary file 1 — Figure S1. Survival according to the number of genomic abnormalities (SV + CNV) detected in the acute myeloid leukaemia (AML) bed file. [file BJH-208-1232-s005.tiff]
