# Supplementary material for: Optical genome mapping detects cryptic high‐risk and targetable abnormalities in adult AML
Source: Br J Haematol. 2026 Feb 1;208(4):1232–9. doi: 10.1111/bjh.70349 (PMC13071487; doi:10.1111/bjh.70349)
Supplement: Supplementary file 2 — Table S1. Acute myeloid leukaemia (AML) bed file set of 109 myeloid genes and 8 regions of interest. [file BJH-208-1232-s002.docx]

**Supplementary Table S1:** AML bed file Set of 109 myeloid genes and 8 regions of interest

| \| ABL1 \| GNAS \| PIGA \| \| --- \| --- \| --- \| \| AFDN \| HOXA9 \| PML \| \| AFF1 \| HRAS \| PPM1D \| \| ANKRD26 \| IDH1 \| PRDM16 \| \| ASXL1 \| IDH2 \| PRPF8 \| \| BCOR \| IL6 \| PTPN11 \| \| BCORL1 \| JAK2 \| RAD21 \| \| BCR \| KAT6A \| RANBP17 \| \| CALR \| KDM5A \| RARA \| \| CBFA2T3 \| KIT \| RARB \| \| CBFB \| KMT2A \| RASSF1 \| \| CBL \| KRAS \| RB1 \| \| CBLB \| LAMP1 \| RBM15 \| \| CBLC \| LUC7L2 \| RPN1 \| \| CCND1 \| MECOM \| RPS14 \| \| CCR7 \| MLF1 \| RUNX1 \| \| CEBPA \| MLLT1 \| RUNX1T1 \| \| CHIC2 \| MLLT10 \| SAMD9 \| \| CREBBP \| MLLT3 \| SAMD9L \| \| CSF3R \| MNX1 \| SETBP1 \| \| CUX1 \| MPL \| SF3B1 \| \| DDX41 \| MRTFA \| SH2B3 \| \| DEK \| MYB \| SMC1A \| \| DNMT3A \| MYC \| SMC3 \| \| EGR1 \| MYH11 \| SRP72 \| \| ELL \| NF1 \| SRSF2 \| \| EGR1 \| NPM1 \| STAG2 \| \| ETNK1 \| NRAS \| TERC \| \| ETV6 \| NSD1 \| TERT \| \| EZH2 \| NUP214 \| TET2 \| \| FGFR1 \| NUP98 \| TP53 \| \| FIP1L1 \| PCM1 \| U2AF1 \| \| FLT1 \| PDGFRA \| U2AF2 \| \| FLT3 \| PDGFRB \| WT1 \| \| FUS \| PHF6 \| ZBTB16 \| \| GATA2 \| PICALM \| ZRSR2 \| \| GLIS2 \|  \| | \| 3q \| \| --- \| \| del(5q)/-5 \| \| del(7q)/-7 \| \| 8 \| \| 12 \| \| del(17p)/-17 \| \| del(20q) \| \| Y \| |
| --- | --- | --- | --- | --- | --- | --- | --- | --- | --- | --- | --- | --- | --- | --- | --- | --- | --- | --- | --- | --- | --- | --- | --- | --- | --- | --- | --- | --- | --- | --- | --- | --- | --- | --- | --- | --- | --- | --- | --- | --- | --- | --- | --- | --- | --- | --- | --- | --- | --- | --- | --- | --- | --- | --- | --- | --- | --- | --- | --- | --- | --- | --- | --- | --- | --- | --- | --- | --- | --- | --- | --- | --- | --- | --- | --- | --- | --- | --- | --- | --- | --- | --- | --- | --- | --- | --- | --- | --- | --- | --- | --- | --- | --- | --- | --- | --- | --- | --- | --- | --- | --- | --- | --- | --- | --- | --- | --- | --- | --- | --- | --- | --- | --- | --- | --- | --- | --- | --- | --- |
