# Supplementary material for: Optical genome mapping detects cryptic high‐risk and targetable abnormalities in adult AML
Source: Br J Haematol. 2026 Feb 1;208(4):1232–9. doi: 10.1111/bjh.70349 (PMC13071487; doi:10.1111/bjh.70349)
Supplement: Supplementary file 3 — Table S2. Targeted next‐generation sequencing of 58 genes recurrently mutated in myeloid neoplasms. [file BJH-208-1232-s003.docx]

**Supplementary Table S**2 : Targeted next-generation sequencing of **58 genes recurrently mutated in myeloid neoplasms**

Genomic DNA was extracted from bone marrow using standard procedures and sequenced on an Illumina NextSeq550Dx platform with the Magnis SureSelect XT HS capture panel (Agilent, Santa Clara, CA, USA). This panel covers the coding regions and adjacent splicing sites (-2 to +2) of 58 genes frequently mutated in myeloid neoplasms (Supplemental 1B), with a minimum sequencing depth of 1000X. Raw NGS data were processed using multiple variant callers (Mutect2, Varscan2, Lofreq, and Vardict), with results aggregated through an in-house pipeline. Variants were interpreted based on minor allele frequencies (MAF) from the GnomAD database, excluding polymorphisms with a MAF>0.02, and assessed for variant allele frequencies (VAF), clinical relevance (COSMIC), and protein impact. All variants were manually reviewed using the Integrative Genomics Viewer and annotated following the Human Genome Variation Society guidelines.

| *ANKRD26* | MPL |
| --- | --- |
| ASXL1 | MYC |
| ASXL2 | NFE2 |
| BCOR | NPM1 |
| BCORL1 | NRAS |
| CALR | PHF6 |
| CBL | PPM1D |
| CCND2 | PTEN |
| CEBPA | PTPN11 |
| CSF3R | RAD21 |
| CUX1 | RIT1 |
| DDX41 | RUNX1 |
| DHX15 | SAMD9 |
| DNMT3A | SAMD9L |
| ETNK1 | SETBP1 |
| ETV6 | SF3B1 |
| EZH2 | SH2B3 |
| FLT3 | SMC1A |
| GATA1 | SMC3 |
| GATA2 | SRSF2 |
| GNAS | STAG2 |
| GNB1 | TET2 |
| IDH1 | TERC |
| IDH2 | TERT |
| IKZF1 | TP53 |
| JAK2 | U2AF1 |
| KDM6A | UBA1 |
| KIT | WT1 |
| KRAS | ZRSR2 |
