# Supplementary material for: Optical genome mapping detects cryptic high‐risk and targetable abnormalities in adult AML
Source: Br J Haematol. 2026 Feb 1;208(4):1232–9. doi: 10.1111/bjh.70349 (PMC13071487; doi:10.1111/bjh.70349)
Supplement: Supplementary file 4 — Table S3. Discrepancies between karyotype and optical genome mapping (OGM) and prognostic impact. [file BJH-208-1232-s004.docx]

**Supplementary Table S3 : discrepancies between karyotype and OGM and prognostic impact**

| **UPN** | **Karyotype** | **Mutations ELN 2022** | **ELN 2022 before OGM** | **Additionnal CA by OGM** | **FISH or PCR confirmation** | **Comments** | **number of additional ELN abnormalities** | **ELN 2022 post OGM** |
| --- | --- | --- | --- | --- | --- | --- | --- | --- |
| **additionnal ELN 2022 anomalies** | | | | | | | | |
| 1 | 47,XY,+8[28]/46,XY[2] | none | intermediate | ogm[GRCh38] t(9;14)(q34.3;q32.13) | confirmed FISH: t(9;14)(wcp9+,wcp14+)(wcp14+,wcp9+)[2] | one more anomaly | 1 | intermediate |
| 2 | 46,XX,der(7)t(7;13)(q33;q31)[29]/46,XX[1] | none | intermediate | ogm[GRCh38] t(6;9)(p22.3;q34.13)*(DEK::NUP14)* | confirmed - PCR | ***DEK::NUP214*** | *1* | **adverse** |
| 3 | 46,XY,t(20;21)(q12;q22) [25] | *BCOR* | adverse | t(8;21;12)(p21.3;q22.12;q24.31)(*RUNX1::RILPL1*),20q11.21q13.13(32205417_50944958)x1~2 | confirmed FISH | Identified of a complex rearrangment | 1 | adverse |
| 4 | 48,XY,+8,+i(8)(q10)[25] | *NPM1* | favorable | ogm[GRCh38] (12)x3 | confirmed FISH: nuc ish (xce12x3)[24/100] | **CK** | 1 | **adverse** |
| 5 | 47,XY,+13[24]/46,XY[1] | *bZIP inframe CEBPA* | favorable | ogm[GRCh38] 1p36.21p35.2(12829120_30497669)x1 | NA | one more anomaly | 1 | favorable |
| 6 | 47,XY,+8[18]/46XY[4] | *NPM1* | favorable | ogm[GRCh38] 24q11.223(23008278_23733292)x0~1 | confirmed FISH: nuc ish (xceXx1, xceYx0)[8/100]) | one more anomaly | 1 | favorable |
| 7 | 47,XY,+8[25] | none | intermediate | ogm[GRCh38]t(X;Y)(q28;q12), t(5;11)(q35.3;p15.4)*(NUP98::NSD1)* | confirmed FISH. ish t(X;Y)(wcpX+,wcpY+;wcpY+,wcpX+) and PCR | ***NUP98::NSD1,* CK** | *2* | **adverse** |
| 8 | 46,XX,del(21)(q21q22)[5]/46,XX[15] | *SRSF2* | adverse | ogm[GRCh38] 20q12q13.12(42846380_44744279)x1 | confirmed FISH del(20q)(PTPRT-)[9/100] | one more anomaly | 1 | adverse |
| 9 | 46,XY,del(20)(q11q13)[20] | none | intermediate | ogm[GRCh38] Yq11.23q12(26574528_57212132)x0 | NA | one more anomaly | 1 | intermediate |
| 10 | 46,XX,add(7)(q22)[12]/46,XX,del(7)(q22q36)[4]/46,XX[4] | none | intermediate | ogm[GRCh38] 2p25.3(1615705_3017923)x1,(4)x1,(22)x3 ; der(7)t(2;7)(p16.2;q22.1) | confirmed FISH. ish der(7)t(2;7)[3] | **CK** | 3 | **adverse** |
| 11 | 45,X,-Y[20] | none | intermediate | ogm[GRCh38] t(5;11)(q35.3;p15.4)*(NUP98::NSD1)* | confirmed - PCR | ***NUP98::NSD1*** | *1* | intermediate |
| 12 | 47,XX,+11[3]/46,XX[17] | none | intermediate | ogm[GRCh38] 6q13q16.1(75175374_92868167)x1 | NA | one more anomaly | 1 | intermediate |
| 13 | 46,XX,del(7)(q22q36)[20] | none | intermediate | ogm[GRCh38] der(7)t(3;7)(q26.2;q21.2) | confirmed FISH der(7)t(3;7)(wcp7+,MECOM+,wcp7-) | **3q abnormality** | 1 | **adverse** |
| 14 | 48,XX,+4,+13[8]/46,XX[7] | *RUNX1, SF3B1* | adverse | ogm[GRCh38] (19)x1,(20)x1 | confirmed FISH : (xce19x1)[12/100],(xce20x1)[7/100] | **CK** | 2 | adverse |
| 15 | 46,XY,t(6;21)(q21;q22)[12]/45,sl,-Y[4]/46,XY[4] | none | intermediate | ogm[GRCh38] (8)x3, ins(11;?)(q23.3;?) | confirmed FISH :(xce8x3)[17/100] / confirmed PCR (MLL-PTD) | **CK, *KMT2A-PTD*** | 1 | **adverse** |
| 16 | 47,XY+8[5]/46,XY[15] | *ASXL1* | adverse | ogm[GRCh38] Yq11.23q12(26574528_57212132)x0 | NA | one more anomaly | 1 | adverse |
| 17 | 46,XX,t(12;22)(p13;q12)[11]/46,XX[9] | *SRSF2* | adverse | ogm[GRCh38] 20q12(42562662_42668561)x1 | NA | one more anomaly | 1 | adverse |
| 18 | 45,X,-X[3]/46,XX[17] | *FLT3-ITD* | intermediate | ogm[GRCh38] t(5;11)(q35.3;p15.4)*(NUP98::NSD1)* , (8)x3 | confirmed FISH (xce8x3)[36/100])/confirmed - PCR | ***NUP98::NSD1****,* **CK** | *2* | **adverse** |
| 19 | 46,XY,+1,der(1;7)(q10;p10)[20] | *RUNX1, U2AF1, BCOR, STAG2* | adverse | ogm[GRCh38] Yq11.23q12(26563807_57212132)x0 | confirmed FISH | **CK** | 1 | adverse |
| 20 | 46,XX,del(7)(q22q36)[9]/46,sl,del(20)(q12q13)[11] | SRSF2 | adverse | ogm[GRCh38] 5p14.3p13.3(18527423_30713952)x1,13q21.2q21.32(61485268_65630764)x1,18q22.1q22.2(64596530_69799692)x1,(22)x3 | .nuc ish (DLEU1, D13S25,D13S1825)x1[15/200],(BCL2x1)[8/100] | **CK** | 4 | adverse |
| 21 | 47,XY,+13[6]/46,XY[14] | *ASXL1,CEBPA no bZIP, RUNX1, SRSF2* | adverse | ogm[GRCh38] fus(12;12)(p13.32;p13.2) | confirmed FISH: (ETV6x2)(5'ETV6 sep 3'ETV6 x1)(45/100) | one more anomaly | 1 | adverse |
| 22 | 47,XY,+8[12]/46,XY[8] | *RUNX1, ASXL1* | adverse | ogm[GRCh38] (17)x1 | confirmed FISH : (xce17x1)[13/100] | **MK** | 1 | adverse |
| 23 | 46,XX,del(20)(q11q13)[18]/46,XX[2] | none | intermediate | ogm[GRCh38] 7q22.1(100292186_102691012)x1 | NA | one more anomaly | 1 | intermediate |
| 24 | 47,XY,+19[10]/48,sl,+13[7]/46,XY[3] | *ZRSR2* | adverse | ogm[GRCh38] t(4;8)(q34.3;q24.13) | confirmed FISH | **CK** | 1 | adverse |
| 25 | 46,XY,+1,der(1;7)(q10;p10)[10] | *EZH2, ASLX1, RUNX1, ZRSR2* | adverse | ogm[GRCh38] Yq11.23q12(26551167_57212132)x0,(19)x1 | confirmed FISH:(BCL3x1)[19/100],(xceYx0)[7/100] | **CK** | 2 | adverse |
| 26 | 46,XY,t(11;21)(q24;q21)[10] | none | intermediate | ogm[GRCh38](7)x1,ins(11;?)(q23.3;?) | confirmed FISH: (xce7x1)[12/100]/confirmed PCR (MLL-PTD) | **MK, *KMT2A-PTD*** | 1 | **adverse** |
| **additionnal no ELN2022 anomalies** | | | | | | | | |
| 27 | 46,XY,add(16)(p1?1)[11]/46,XY[9] | *NPM1, FLT3-ITD* | intermediate | ogm[GRCh38] t(2;16)(p23.3;p11.2) | .ish t(2;16)(p2?4;p1?2)(MYH11+;CBFB+)[3].nuc ish (MYH11,CBFB)x2 [98/100] | identified derivative chromosome | 0 | intermediate |
| 28 | 46,XX,del(7)(q35q36)[3]/46,XX[17] | *RUNX1, ASXL1, TP53, U2AF1* | adverse | ogm[GRCh38] ins(11;?)(q23.3;?) | confirmed PCR (MLL-PTD) | ***KMT2A-PTD*** | 0 | adverse |
| 29 | 47,XX,+r[6]/46,XX[14] | *NPM1* | favorable | ogm[GRCh38] (8)x3 | confirmed FISH : r(8)(xcp8+)[4] | identified ring chromosome | 0 | favorable |
| 30 | 47,XX,t(10;17;17)(q22;q25;q11),+11[25] | none | intermediate | Putative Gene Fusion*: KAT6B::USP36* | NA | Identified partner gene | 0 | intermediate |
| 31 | 47,XX,+8[4]/46,XX[16] | FLT3-ITD | intermediate | ogm[GRCh38] ins(11;?)(q23.3;?) | confirmed PCR (MLL-PTD) | ***KMT2A-PTD*** | 0 | intermediate |
| 32 | 46,XX,del(7)(q22q36)[18]/46,XX[2] | *bZIP inframe CEBPA* | favorable | ogm[GRCh38] ins(11;?)(q23.3;?) | confirmed PCR (MLL-PTD) | ***KMT2A-PTD*** | 0 | favorable |
| 33 | 47,XY,+11[20] | *BCOR* | adverse | ogm[GRCh38] ins(11;?)(q23.3;?) | confirmed PCR (MLL-PTD) | ***KMT2A-PTD*** | 0 | adverse |
| 34 | 46,XY,del(7)(q21q36)[7]/46,XY[13] | *RUNX1, BCOR* | adverse | ogm[GRCh38] ogm[GRCh38] fus(7;7)(q21.2;q36.3) Putative Gene Fusion CDK6::MNX1 | NA | Identified of a complex rearrangment | 0 | adverse |
| 35 | 46,XY,add(9)(p24)[6]/47,XY,+8[2]/ 46,XY[22] | *NPM1, FLT3-ITD* | intermediate | ogm[GRCh38] 9p11.2p11.1(41534477_42450779)amp,9q21.11(65594150_66250439)amp | NA | identified derivative chromosome | 0 | intermediate |
| 36 | 45,X,-Y,del(9)(q22q33)[8]/46,XY[12] | *FLT3-ITD, ZRSR2* | adverse | ogm[GRCh38] ins(11;?)(q23.3;?) | confirmed PCR (MLL-PTD) | ***KMT2A-PTD*** | 0 | adverse |
| 37 | 46,XY,t(1;12)(p12;p13)[5] | *SF3B1* | adverse | Putative Gene Fusion : *FAM102B::WBP11* |  | Identified partner gene | 0 | adverse |
| **discordant anomalies** | | | | | | | | |
| 38 | 47,XY,+mar[2]/46,XY[58] | *RUNX1* | adverse | ogm[GRCh38] (X,Y)x1,(1-22)x2 | NA |  |  | adverse |
| 39 | 45,XY,-15[5]/46,XY[20] | *NPM1* | favorable | ogm[GRCh38] (X,Y)x1,(1-22)x2 | confirmed FISH : nuc ish (D15Z1x1)[23/200] |  |  | favorable |
| 40 | 46,XX,del(7)(q31q36)[8]/46,XX[22] | *ASXL1, RUNX1, BCOR* | adverse | ogm[GRCh38](1-22,X)x2 | confirmed FISH : nuc ish (xce7x2,KMT2Ex2,CUL1x1,EZH2x1) [13/100] |  |  | adverse |
| 41 | 46,XY,dic(4;8)(q35;p11)[4]/47,XY,+8[2]/46,XY[19] | *NPM1, FLT3-ITD* | intermediate | ogm[GRCh38] (8)x3 | confirmed FISH: .ish der(4)(MYC+)[1].nuc ish (MYCx3,xce8x2)[10/100] |  |  | intermediate |
| 42 | 46,XX,del(12)(p13p12)[6]/46,sl,t(3;9)(p21;q33)[2]/46,XX[12] | *ASLX1* | adverse | ogm[GRCh38] (1-22,X)x2 | nuc ish (5'ETV6x2),(3'ETV6x1),(5'ETV6 con 3'ETV6x1)[22/200] |  |  | adverse |
| 43 | 46,XY,del(11)(q13q23)[5]/46,XY,del(9)(q22q34)[2]/46,XY[13] | *bZIP inframe CEBPA* | favorable | ogm[GRCh38] 11q14.1q23.2(80240346_113197401)x1~2 | NA |  |  | favorable |
| 44 | 47,XY,+8[4]/46,XY[16] | *ASXL1, EZH2, SRSF2* | adverse | ogm[GRCh38] (X,Y)x1,(1-22)x2 | confirmed FISH: .nuc ish (xce8x3)[8/100] |  |  | adverse |
| 45 | 92,XXXX,der(7)t(7;11)(q31;q21)x2[16]/46,XX[4] | *EZH2* | adverse | ogm[GRCh38]der(7)t(7;11)(q31.31;q22.1) | confirmed FISH |  |  | adverse |
| 46 | 47,XY,+13[2]/46,XY[23] | *RUNX1, SF3B1* | adverse | ogm[GRCh38] Yq11.23q12(26574528_57212132)x0~1 | .nucish (DLEU1, D13S25,D13S1825)x3[6/100],(RP11-214M24)x0[7/100] |  |  | adverse |
| 47 | 47,XY,+mar[3]/46,XY[37] | *NPM1* | favorable | ogm[GRCh38](X)x1,(1-22)x2 | .nuc ish(xceXx1,xceYx0)[16/100] |  |  | favorable |
| 48 | 47,XY,+19[20] | *STAG2* | adverse | ogm[GRCh38] (22)x3 | .ish 19(wcp19+),+22(wcp22+)[4] |  |  | adverse |

ELN: European Leukemia Net, OGM: Optical Genome Mapping, FISH: fluorescence in situ hybridisation, PCR: polymerase Chain Reaction, CA: chromosomal abnormalities, CK: Complex Karyotype, MK: Monosomal Karyotype, NA: Not applicable
