# Supplementary material for: Optical genome mapping detects cryptic high‐risk and targetable abnormalities in adult AML
Source: Br J Haematol. 2026 Feb 1;208(4):1232–9. doi: 10.1111/bjh.70349 (PMC13071487; doi:10.1111/bjh.70349)
Supplement: Supplementary file 5 — Table S4. Number and type of abnormalities, focused on ‘acute myeloid leukaemia (AML) bed file’. [file BJH-208-1232-s001.docx]

**Supplementary Table S4:** Number and type of abnormalities, focused on “AML bed file”.

|  | **Number of patients by type of abnormalities** | | |
| --- | --- | --- | --- |
| **Number of abnormalities** | *SV* | *CNV* | **SV+CNV** |
| 0 | *42* | *8* | 4 |
| 1 | *28* | *50* | 26 |
| 2 | *14* | *18* | 23 |
| 3 | *1* | *6* | 22 |
| 4 | *1* | *4* | 7 |
| 5 | *1* | *1* | 3 |
| 6 | *0* | *0* | 2 |
| Mean | 0,78 | 1,44 | 2,14 |
| Median | 1 | 1 | 2 |
| SD | 0,969 | 0,996 | 1,345 |
| min-max | 0-5 | 0-5 | 0-6 |
| q1-q3 | 0-1 | 1-2 | 1-3 |

SV: structural variants, CNV: copy number variation, SD: standard deviation, IQR: Inter-Quartile Range

4 patients were excluded due to background noise on the CNVs
